# Adverse maternal outcomes among women who gave birth at public hospitals in eastern Ethiopia: a cross-sectional study

**DOI:** 10.3389/fgwh.2025.1569815

**Published:** 2025-03-28

**Authors:** Masresha Leta, Abera Kenay Tura, Haymanot Mezmur, Kasiye Shiferaw, Nega Assefa

**Affiliations:** ^1^ Department of Midwifery, Harar Health Science College, Harar, Ethiopia; ^2^ School of Nursing, College of Health and Medical Sciences, Haramaya University, Harar, Ethiopia; ^3^ Department of International Public Health, Liverpool School of Tropical Medicine, Liverpool, United Kingdom; ^4^ Department of Obstetrics and Gynaecology, University Medical Centre Groningen, Groningen, Netherlands; ^5^ School of Midwifery, College of Health and Medical Sciences, Haramaya University, Harar, Ethiopia

**Keywords:** adverse maternal outcome, adverse maternal in eastern Ethiopia, pregnancy outcomes, obstetrics complications, predictors

## Abstract

**Background:**

An adverse maternal outcome, such as anemia, postpartum hemorrhage, and postpartum eclampsia, poses a significant risk to women. While studies on the burden of adverse maternal outcomes have been conducted in various countries, including Ethiopia, many predictors beyond obstetric factors have not been fully explored. This study aimed to determine the magnitude and factors associated with adverse maternal outcomes among women who gave birth at selected public hospitals in eastern Ethiopia.

**Methods:**

A hospital-based cross-sectional study was conducted among 2,608 randomly selected women who gave birth in six public hospitals in eastern Ethiopia from November 2023 to March 2024. Data were collected through face-to-face interviews and clinical chart reviews. Factors associated with adverse maternal outcomes were identified using bivariable and multivariable robust Poisson regression analyses. Adjusted relative risk (ARR) with a 95% confidence interval (CI) was used to report the strength of the association. The variables with a *p*-value of <0.05 were considered statistically significant.

**Results:**

The magnitude of adverse maternal outcomes was 15.68% (95% CI: 14.70%–16.66%). A poor wealth index (ARR = 4.41; 95% CI: 3.46–5.62), having danger signs at admission (ARR = 1.86; 95% CI: 1.18–2.91), alcohol use during pregnancy (ARR = 1.86; 95% CI: 1.32–2.62), duration of labor ≥24 h (ARR = 1.69; 95% CI: 1.00–2.85), and maternal age greater than 35 years (ARR = 1.39; 95% CI: 1.03–1.86) increased the risk of adverse maternal outcomes. In contrast, folic acid intake during pregnancy (ARR = 0.47; 95% CI: 0.38–0.57), having partner support (ARR = 0.70; 95% CI: 0.59–0.83), and spontaneous vaginal delivery (ARR = 0.58; 95% CI: 0.49–0.68) reduced the risk of adverse maternal outcomes.

**Conclusion:**

One in six women who gave birth in eastern Ethiopia experienced adverse maternal outcomes. This rate was determined to be moderate when compared to the WHO projections for lower- and middle-income countries and better than the higher averages reported by the WHO. Targeted intervention programs, such as targeted education and empowerment programs, and the strengthening of the community health worker program would help address socioeconomic disparities and improve early detection and management of danger signs during pregnancy, which would aid in averting the occurrence of adverse outcomes.

## Introduction

An adverse maternal outcome is any pregnancy-related condition that results in maternal morbidity, potentially life-threatening conditions, maternal near misses [an event where a pregnant woman experiences severe complications during pregnancy, childbirth, or within 42 days following the termination of pregnancy but survives these life-threatening condition (1)], or maternal deaths during or following childbirth (2). The following can be considered an adverse maternal outcome: intensive care init (ICU) admission, anemia, length of hospital stay >48 h for a vaginal birth and >72 h for cesarean section (CS) delivery, pregnancy-related complications [postpartum hemorrhage (PPH), shock, uterine rupture, and endometritis], perineal tear, cervical tear, blood transfusion related to pregnancy**,** postpartum eclampsia, surgical site infections after CS [an infection that occurs at the site of the surgical incision within 30 days following the procedure (3)], and maternal death (4–6).

Adverse maternal outcomes represent a critical public health issue, particularly in low- and middle-income countries (LMICs) where healthcare resources are often limited since the incidence is significantly higher compared to higher-income countries (7). A key component of these outcomes is the need for ICU admission, which serves as an indicator of severe maternal health complications. This necessity is often linked to conditions such as anemia, pregnancy-induced hypertension, and PPH. Admission danger signs [specific symptoms or conditions that indicate a potential risk to the health of the pregnant woman or her fetus (8, 9)], such as hypertensive disorders, are the most prevalent cause of ICU admissions (10). These complications not only lead to increased maternal mortality rates but also result in prolonged hospital stays, averaging 1.3–6.6 days for vaginal deliveries and 2.5–9.3 days for cesarean sections (CSs) (11).

Furthermore, surgical site infections (SSIs) following cesarean deliveries exacerbate the situation, contributing to serious complications and extended recovery times. The prevalence of SSIs varies significantly by region, with notable rates in Africa (11.91%), Ethiopia (12.32%), and North America (3.87%) (12, 13). Another significant concern is postpartum anemia, which affects 50%–80% of mothers in developing countries and is often a consequence of blood loss during delivery or inadequate iron intake, frequently requiring blood transfusions (14).

Despite global efforts to reduce maternal mortality, many women still face life-threatening conditions during and after childbirth (15). The World Health Organization (WHO) estimated that there were approximately 287,000 maternal deaths worldwide in 2020, with sub-Saharan Africa reporting a maternal mortality rate of 542 deaths per 100,000 live births (16, 17). Alarmingly, nearly 75% of these deaths were attributed to preventable complications such as severe bleeding, infections, and hypertension during pregnancy. This underscores the urgent need for improved healthcare interventions to address these interconnected challenges and enhance maternal health outcomes globally (18).

Adverse maternal outcomes have significant impacts on economic stability, family dynamics, national healthcare systems, and obstetrical health (19). Adverse outcomes lead to high medical expenses, particularly in low-income settings, straining family finances (20). For example, maternal morbidity in the USA can cost up to $86 billion annually (21). In low-income countries, maternal deaths can reduce household income by as much as 20%, destabilizing families and affecting child care and household management (22).

At the national level, maternal morbidity and mortality increase the burden on healthcare systems, especially in resource-limited settings, leading to greater demands for services (23). In addition, women who experience severe complications face increased risks in future pregnancies (24); for instance, the WHO reported that such women are 2–4 times more likely to develop cardiovascular diseases later in life (25). Thus, the study's findings could contribute to addressing these challenges by identifying specific risk factors that influence maternal health.

Despite Ethiopia's efforts to implement various strategies to improve maternal health, adverse maternal outcomes remain high due to multifaceted factors. Various factors influence maternal health outcomes in Ethiopia, including healthcare-related, socioeconomic, and cultural factors. Inadequate healthcare infrastructure, such as the availability of skilled birth attendants and quality maternal care services, can lead to complications during childbirth, contributing to high rates of maternal morbidity and mortality. While some studies in Ethiopia have examined adverse maternal outcomes, many of them only focus on obstetrical outcomes and neglect socioeconomic and behavioral factors such as alcohol intake. Studies have shown that even moderate alcohol use during pregnancy can lead to significant health issues for the mother (26). Furthermore, a poor socioeconomic status significantly influences the health behavior of the mother after birth (27). This narrow focus restricts a holistic understanding of the complexities of maternal health challenges (28–30). However, this study not only examines obstetric outcomes but integrates healthcare-related factors with socioeconomic and cultural dimensions since little is known about how such factors are associated with adverse maternal outcomes in Ethiopia, particularly in our study area. This study aims to fill those gaps by integrating a broader range of factors. Comprehending these factors contributes to the country's initiatives to decrease adverse maternal outcomes. It empowers healthcare providers to offer treatment and prevention measures for all pregnant women since every pregnancy by itself is a risk, thus ensuring that every pregnant woman receives appropriate care. This research aimed to determine the magnitude of, and factors associated with, adverse maternal outcomes among women who gave birth in selected public hospitals in eastern Ethiopia (31).

## Methods

### Study design and setting

This was an institutional-based cross-sectional study conducted from 1 November 2023 to 31 March 2024 in six selected public hospitals across the Oromia, Somali, and Harari regional states and the Dire Dawa administration in eastern Ethiopia. Hiwot Fana Comprehensive Specialized Hospital and Jugal General Hospital in the Harari Region, Bisidimo and Haramaya General Hospitals in the Oromia Region, and Dil Chora Referral Hospital and Sabian General Hospital in the Dire Dawa administration were randomly selected.

### Population and sampling

All women who came to the selected hospitals for labor and delivery services and gave live birth or stillbirth were considered for the study. Data from the selected hospitals indicated that between 10,000 and 13,000 deliveries had been conducted in the 6 months before data collection. This study was part of a PhD research project with multiple objectives. Separate sample sizes were initially calculated for adverse maternal outcomes (2,326) and adverse fetal outcomes (2,608). The larger sample size calculated for adverse fetal outcomes was chosen to ensure adequate statistical power to detect significant associations. To strengthen the analysis, we decided to use this larger sample size to examine the magnitude of, and factors associated with, adverse maternal outcomes as well. The sample size was allocated proportionally based on the caseload of each hospital. The calculated sample sizes were as follows: 914 for Haramaya Hospital, 522 for Hiwot Fana Specialized Comprehensive Hospital, 483 for Dil Chora Hospital, 268 for Sabian General Hospital, 260 for Jugal Hospital, and 161 for Bisidimo Hospital.

Based on this information, systematic random sampling was employed to select respondents, calculating *N*/*n* = 12,240/2,608 = 4.69–5. Every fifth woman was selected after the first woman was randomly chosen. Initially, we assigned numbers to the first five mothers who arrived to give birth at the start of the study. From these five, one mother was randomly selected to serve as the starting point. From that point onward, we proceeded systematically, using this selected mother as the reference point, until the required sample size was achieved. Mothers who were ineligible (such as those who gave birth in other hospitals and came for management of complications) or declined to participate were excluded from the study since we were unable to track their status after the mother was referred to other hospitals. In addition, if the mother gave birth at a different facility and later presented at our hospital, it was challenging to accurately identify the factors contributing to the occurrence of the adverse outcomes. Furthermore, those who were transferred for specialized care from these hospitals were also excluded from the study. Subsequently, the next eligible mother undergoing the procedure was randomly selected.

### Study variables and data collection

The study used a structured questionnaire adapted from the literature (32–34) to ensure their relevance and applicability in the Ethiopian context. The adaptation process began by selecting appropriate measuring instruments that aligned with our research objectives and then a pilot test was conducted to assess the feasibility and clarity of the adapted tools. The questionnaire was prepared in English and was translated into the local languages (Amharic, Afan-Oromo, and Af-Somali). A tool validated by the researchers was used for the collection of the data. The tool gathers data on sociodemographic and maternal behavioral factors, past and current obstetric history, and related care characteristics. In addition, we measured perceived stress during pregnancy using the global measure of perceived stress scale, a 14-component scale (35), and anxiety during pregnancy using the Kessler Psychological Distress Scale, comprising 10 items (36). Both stress and anxiety were assessed using a five-point Likert scale, which categorized the participants' experiences as follows: not at all stressful/anxious, a little bit stressful/anxious, moderately stressful/anxious, very stressful/anxious, and extremely stressful/anxious. To simplify the analysis, we implemented a threshold where ratings of 1 and 2 were classified as “not stressful/anxious” (generally considered “No”), while ratings of 3, 4, and 5 were classified as “having stress/anxiety” (generally considered “Yes”), indicating moderate to high levels of stress or anxiety. Similarly, the Turner Support Scale was used to assess partner support. This tool has four components, and each component reflects a different aspect of perceived partner support (37). For intimate partner violence, a standard tool for domestic violence and abuse (38) was used, and it has four components with a four-point Likert scale to quantify the level of violence experienced.

Maternal information such as sociodemographic characteristics, behavioral characteristics, obstetrical characteristics, and professional factors were assessed using interviewer-administered questionnaires. All data collection procedures followed the national infection prevention guidelines (meaning that the methods used to gather data adhered to established protocols aimed at preventing infections). We utilized electronic data collection tools (Kobo toolbox v2024.2.4), and data were collected by six female BSc midwives recruited from outside the selected hospitals. Two MSc midwife supervisors were assigned to oversee the entire data collection process. One day of training was provided for data collectors and supervisors to ensure consistency in the data collection process. The study period starts at the admission of the mother and lasts once the mother is discharged from the hospital. Women who gave birth at home or in other places and those referred to other hospitals were excluded. Data were collected through face-to-face interviews with individuals with a good command of language. A further chart review was conducted for all cases. The information was kept confidential using computer-generated random identification (ID) numbers to create study IDs that were not associated with their names or any other identifying details.

### Data quality assurance

The questionnaire was pretested on 10% of the sample size before data collection in a similar setup at Deder Hospital (a hospital out of the study region) to check the appropriateness of the data collection tool, consistency, and participant selection, and necessary modifications were made accordingly. Data collectors were supervised throughout the data collection period, and regular feedback was provided to them based on the reviewed data to improve data collection quality. Validated tools were used for the collection of the data. The completeness and accuracy of the data were monitored daily. The principal investigator ensured the reliability of the data by randomly reviewing completed questionnaires and monitoring daily data collection activities. During data collection time, real-time checks were conducted to identify errors and ensure immediate corrections.

### Data analysis

The data for each question in the questionnaire were manually reviewed for completeness and consistency before being transferred to Stata 14 for analysis. In this study, we employed a complete case analysis, meaning that observations with any missing data for the variables included in a particular analysis were excluded*.* Descriptive statistics were calculated to characterize the study population using percentages, means, and standard deviation. A wealth index was calculated using principal component analysis (PCA) by considering ownership of assets (such as cars and refrigerators), type of housing, and access to utilities (such as electricity and water) by interviewing the mothers during a survey. A Poisson regression model with robust variance was used to examine the association between adverse maternal outcomes and selected variables. We chose Poisson regression for this analysis because adverse maternal outcomes are count data, representing the number of adverse events experienced by each participant, and Poisson regression is specifically designed to model count data. We used robust variance estimation to address the issue of overdispersion in our data. Overdispersion occurs when the variance of the count data is greater than its mean, violating a key assumption of standard Poisson regression. Variables with a *p*-value < 0.25 in the bivariable analysis were considered for multivariable analysis. Crude risk ratios (CRRs) and adjusted relative risk (ARR) were calculated with 95% confidence intervals (CIs). In our study, we accounted for several potential confounders that could affect the relationship between maternal factors, such as prior birth histories, access to healthcare for antenatal care (ANC) check-ups during the current pregnancy, and other variables. By incorporating these factors into the multivariable model, we aimed to minimize the influence of confounding variables on our ARR estimates, ensuring that our findings represent a true association. Statistically, a significant association was declared at a *p*-value less than 0.05 with a 95% confidence interval. The Akaike information criterion (AIC) method, with a value of 3004.006, and the Bayesian information criterion (BIC) method, with a value of 3021.605, were used for model selection. The fitness of the model was assessed using the Hosmer–Lemeshow goodness-of-fit test. The presence of multicollinearity was checked using the variance inflation factor (VIF) before fitting to the model and the variables with a VIF < 10 were selected for the final model. We calculated the VIF for each predictor variable related to adverse maternal outcomes and all had VIF values <10.

### Operational definitions of key terms related to adverse maternal outcomes

Adverse maternal outcomes included PPH, hysterectomy, uterine rupture, puerperal sepsis, anemia, bladder trauma, perineal tear, cervical tear, surgical site complications following caesarean or instrumental vaginal delivery, and maternal deaths. If the mother had one or more of the above conditions (recorded on the chart), she was considered to have experienced adverse maternal outcomes (39–41). These adverse outcomes were identified based on clinical diagnoses documented in the maternal chart and were measured based on established obstetrical criteria throughout the study period.

Admission danger signs included hemorrhage, pregnancy-induced hypertension, infection, and obstructed and prolonged labor, or if the mother developed at least one of the nine danger signs stated in the WHO guide list. No danger sign was indicated if no signs developed (42, 43).

Partner support (PS) was measured by an 8-item partner support scale, and if the respondent scored the mean value or above the mean value, they were considered to have received partner support during pregnancy (44).

Intimate partner violence (IPV) was measured to ascertain whether the women experienced physical, psychological, or sexual violence. This was conducted using a 13-item scale (four for psychological violence, six for physical violence, and three for sexual violence), and if the women responded to at least one, the presence of IPV was considered (45).

Stress refers to the physiological and psychological responses that arise when individuals perceive a threat or challenge (46). We measured perceived stress during pregnancy using a 14-component global measure of perceived stress scale (47) and framed a “Yes/No: question to help capture perceived stress during pregnancy.

Anxiety is an emotion characterized by an unpleasant state of inner turmoil that includes feelings of dread over anticipated events (48). We measured anxiety during pregnancy using the Kessler Psychological Distress Scale, which is comprised of 10 items (36), and framed a “Yes/No” question to help capture anxiety during pregnancy.

### Ethical approval and consent to participate

Ethical clearance was obtained from the Institutional Health Research Ethical Review Committee (Ref. no. IHRERC/190/2023), College of Health and Medical Sciences, Haramaya University. The purpose, procedure and duration, possible risks, and benefits of the study were explained and informed consent was obtained from each participant. For women younger than 18 years old, informed consent was obtained from their legal guardians for their protection. In addition, we sought oral assent from the minors themselves, ensuring they understood the study's purpose and procedures in a manner appropriate for their age. All respondents gave their informed consent, and the study was carried out in compliance with the Declaration of Helsinki. The information sheet outlined the study's title, objectives, procedures, risks, and benefits of participation. Respondents were informed of their right to decline participation or withdraw from the study at any time.

## Results

### Sociodemographic characteristics of the respondents

A total of 2,608 women were enrolled in the study, with a 100% response rate. As shown in Table 1, the majority (84.32%) of the women were aged 15–24 years old, with a mean age of 26 (+5.1 years). The majority of respondents were urban residents (60.43%). Approximately three-fourths of the study respondents (73.61%) were housewives, and the majority of them (98.08%) were married. Those who attended primary education accounted for 43.79%, and most (30.37%) belonged to the lowest wealth index classification.

**Table 1:** Sociodemographic characteristics of the women who gave birth at selected public hospitals in eastern Ethiopia in 2024 (*n* = 2,608).

Variable	*N*	Percentage
Age (years)		
15–24	2,199	84.32
≥25	409	15.68
Residency		
Urban	1,576	60.43
Rural	1,032	39.57
Ethnicity		
Oromo	2,015	77.26
Amhara	379	14.53
Gurage	107	4.10
Others^a^	107	4.10
Occupation		
Government employee	212	8.13
Self-employed	244	9.36
Housewife	1,920	73.61
Others^b^	232	8.9
Religion		
Muslim	2,068	79.29
Christian	540	20.71
Educational status		
No formal education	623	23.89
Primary education	1,142	43.79
Secondary education	544	20.86
Technical education	110	4.22
Higher education	189	7.25
Educational status of the husband		
No formal education	359	13.77
Primary education	977	37.46
Secondary education	720	27.61
Technical education	185	7.09
Higher education	367	14.07
Wealth index (household)		
Poorest	792	30.37
Poor	392	15.03
Middle	449	17.22
Rich	534	20.48
Richest	441	16.91

^a^ Others: Harari, Somali.

^b^ Others: NGO, daily laborer.

### Obstetrics-related characteristics of the respondents

Among the study respondents, as shown in Table 2, the majority (49.62%) of women were pregnant more than once, with 45.55% having a birth spacing of 24–59 months. Approximately 6.6% of these women had a history of complications during past pregnancies, either with their baby or themselves. Furthermore, 71.62% of the respondents had received antenatal care, with 56.15% starting in the first trimester. Regarding danger signs upon admission, 20.17% of the women experienced them, with 24.73% being diagnosed with pregnancy abnormalities, including APH, PIH, and amniotic fluid abnormalities. Of all the respondents, 27.22% were referred from private clinics and other government health facilities, with the majority (54.37%) taking more than 30 min to reach the health facilities. Approximately 87.58% of the women went into labor spontaneously, with 81.17% having a total labor duration of less than 12 h.

**Table 2:** Obstetrics-related characteristics of women who gave birth at selected public hospitals in eastern Ethiopia in 2024 (*n* = 2,608).

Variable	Frequency	%
Gravida		
Primigravida	814	31.22
Multigravida	1,294	49.61
Grand multigravida	500	19.17
Indexed pregnancy		
0–23 months	287	11
24–59 months	1,188	45.55
≥60 months	319	12.23
Complication in past pregnancy		
Yes	172	6.60
No	1,622	62.19
Antenatal care (ANC) follow-up for this pregnancy		
Yes	1,870	71.70
No	738	28.30
Initiation of ANC		
First trimester	1,049	40.22
Second trimester	638	24.46
Third trimester	181	6.94
Danger sign at admission		
Yes	601	23.04
No	2,007	76.96
Having labor complications		
Yes	602	23.08
No	2,006	76.91
Referral status		
Yes	710	27.22
No	1,898	72.78
Time to reach health facilities from the referral point (*n* = 710)		
<30 min	324	45.63
30–60 min	276	38.87
≥60 min	110	15.5
Onset of labor		
Spontaneous	2,284	87.58
Induced	324	12.42
Labor duration		
<12 h	2,117	81.17
12–24 h	401	15.38
≥24 h	50	1.92

### Social and behavior-related characteristics of respondents

Regarding the history of substance use, as shown in Table 3, 18.37%, 5.18%, and 1.04% used khat, alcohol, and either smoked cigarettes or shisha during their current pregnancy, respectively. In this study, a total of 58.63% of respondents did not receive any partner support during their pregnancy. Approximately 43.60% and 49.50% of the study respondents experienced stress and anxiety during their pregnancy, respectively. Since stress and anxiety were assessed regarding the mothers’ experience in the previous 4 weeks before birth and the interview was conducted after she gave birth, they can be considered predictive factors for adverse maternal outcomes.

**Table 3:** Social and behavior-related characteristics of the study respondents during pregnancy in eastern Ethiopia in 2024.

Variable	Frequency	Percentage
Khat chewing		
Yes	479	18.37
No	2,129	81.63
Alcohol intake		
Yes	135	5.18
No	2,473	94.82
Cigarette (Shisha) smoking		
Yes	27	1.04
No	2,581	98.96
Partner support		
Yes	1,079	41.37
No	1,529	58.63
Intimate partner violence		
Yes	53	2.03
No	2,555	97.97
Perceived stress		
Yes	1,137	43.60
No	1,471	56.40
Anxiety		
Yes	1,291	49.50
No	1,317	50.50

### Magnitude of adverse maternal outcomes

The magnitude of adverse maternal outcomes was 15.68% (95% CI: 14.70%–16.66%). As shown in Table 4, the most common adverse maternal outcome was anemia (89.97%), followed by postpartum hemorrhage (3.6%). Among those who experienced an adverse maternal outcome, 23.71% and 10.51% had long hospital stays following CS and vaginal birth due to PPH and postpartum eclampsia.

**Table 4:** Adverse maternal outcomes among women attending the study hospitals for delivery care in eastern Ethiopia in 2024.

S. no	Adverse maternal outcome	Frequency	Percentage
1.	Anemia	368	89.97
2.	Postpartum hemorrhage	15	3.6
3.	Pregnancy-induced hypertension (severe pre-eclampsia/eclampsia)	12	2.9
4.	Endometritis	5	1.2
5.	Hysterectomy	5	1.2
6.	Surgical site infection	4	1.13
Total		409	100

### Predictors for adverse maternal outcomes

As shown in Table 5, study respondents in the poor wealth index category, maternal age greater than 35 years, drinking alcohol during pregnancy, spontaneous vaginal delivery, duration of labor lasting longer than or equal to 24 h, having partner support during pregnancy, diagnosed with an admission danger sign, and folic acid intake during pregnancy were significantly associated with adverse maternal outcomes. Maternal age older than 35 years demonstrated a significantly increased risk of adverse maternal outcomes with an ARR = 2.13 (95% CI; 1.52–2.98). In addition, the presence of admission danger signs was associated with increased risk of adverse maternal outcomes, with an ARR = 2.75 (95% CI; 2.26–3.35). Similarly, pregnant women classified in the poor wealth index category had a more than fourfold increase in the risk of adverse maternal outcomes, with an ARR = 3.81 (95% CI; 2.96–4.89). Furthermore, pregnant women who drank alcohol during pregnancy and mothers who had a labor duration of longer than or equal to 24 h demonstrated a significantly increased risk of adverse maternal outcomes with an ARR = 1.86 (95% CI; 1.32–2.62) and an ARR = 1.69 (95% CI; 1.00–2.85), respectively. However, folic acid intake during pregnancy, having partner support during pregnancy, and those experiencing a spontaneous vaginal delivery were associated with a significant reduction in the risk of adverse maternal outcomes with an ARR = 0.47 (95% CI; 0.38–0.57), an ARR = 0.70 (95% CI; 0.59–0.83), and an ARR = 0.58 (95% CI; 0.49–0.68), respectively.

**Table 5:** Predictors of adverse maternal outcomes among women who gave birth at public hospitals in eastern Ethiopia in 2024.

Variable	Adverse maternal outcome		CRR (95% CI)	ARR (95% CI)	*P*-value
	Yes	No			
Age group					
15–24	390	1,809	Ref	Ref	
25–35	76	325	2.81 (1.40–5.67)	0.87 (0.68–1.11)	0.28
36–45	4	4	1.06 (0.85–1.33)	2.13 (1.52–2.98)*	<0.001
Wealth index					
Poorest	77	715	Ref	Ref	
Poor	159	233	4.17 (3.26–5.32)	3.81 (2.96–4.89)*	<0.001
Middle	37	412	0.84 (0.58–1.23)	1.12 (0.77–1.63)	0.53
Rich	109	425	2.09 (1.60–2.75)	1.20 (0.71–2.01)	0.48
Richest	88	353	2.05 (1.54–2.72)	1.32 (0.77–2.26)	0.30
Anxiety					
Yes	164	1,127	0.54 (0.45–0.65)	1.07 (0.68–1.68)	0.76
No	306	1,011	Ref	Ref	
Mode of delivery					
SVD	269	1,476	0.66 (0.56–0.77)	0.58 (0.49–0.68)*	<0.001
CS	201	662	Ref	Ref	
Alcohol use during pregnancy					
Yes	216	97	0.27 (0.13–0.57)	1.86 (1.32–2.62)*	<0.001
No	254	2,041	Ref	Ref	
Duration of labor					
<12 h	400	1,732	1.43 (1.09–1.88)	1.16 (0.89–1.49)	0.25
12–24 h	55	359	Ref	Ref	
≥24 h	15	47	1.85 (1.06–3.22)	1.69 (1.00–2.85)*	0.04
Residency					
Urban	216	1,360	Ref	Ref	
Rural	193	839	0.75 (0.63–0.88)	0.95 (0.79–1.14)	0.58
Partner support during pregnancy					
Yes	269	1,260	2.17 (1.75–2.68)	0.70 (0.59–0.83)*	<0.001
No	201	878	Ref	Ref	
Danger sign at admission					
Present	158	368	2.00 (1.69–2.36)	2.75 (2.26–3.35)*	<0.007
Absent	312	1,770	Ref	Ref	
Folic Acid intake					
Yes	214	1,328	0.46 (0.37–0.56)	0.62 (0.51–0.76)*	<0.001
No	98	228	Ref	Ref	

CRR, cumulative risk ratio; ARR, adjusted relative risk; CS, cesarean section; SVD, spontaneous vaginal delivery.

* Statistical significance.

## Discussion

The objective of this study was to investigate the magnitude of, and factors associated with, the occurrence of adverse maternal outcomes among mothers who gave birth at selected public hospitals. The study found that the prevalence of adverse maternal outcomes was 15.68% (95% CI: 14.70%–16.66%). The study revealed that the main adverse maternal outcome after childbirth was anemia (89.97%), followed by postpartum hemorrhage (3.6%). Among women who experienced an adverse maternal outcome, 23.71% and 10.51% had long hospital stays following CS and vaginal birth, respectively, due to PPH and postpartum eclampsia. This study identified several factors that were significantly associated with adverse maternal outcomes, including poor wealth index classification, maternal age older than 35 years, drinking alcohol during pregnancy, spontaneous vaginal birth, a labor duration of ≥24 h, having partner support during pregnancy, diagnosis with an admission danger sign, and folic acid intake during pregnancy.

The overall prevalence of adverse maternal outcomes was 15.68%, and this was considered to be moderate when compared to the WHO projections for lower- and middle-income countries, reflecting that there are ongoing challenges in maternal health that need attention. This finding is congruent with a study in southern Ethiopia that revealed that the magnitude of PPH was 16.6% (49). Similarly, a systematic review and meta-analysis study conducted in Ethiopia on obstetrics emergencies and adverse maternal and perinatal outcomes showed that the magnitude of adverse maternal outcomes was 15.9% (50).

Comparatively, a study conducted in North Ethiopia reported higher rates of adverse maternal outcomes with 32.6% (anemia and PIH with 24.1% and 20.7%, respectively) (51). This difference could be attributed to variation in the study population in terms of demographic and clinical characteristics, quality of prenatal care, and access to healthcare services, which can greatly affect maternal outcomes.

The results showed that women whose age was greater than 35 years (ARR = 2.13; 95% CI; 1.52–2.98) had adverse maternal outcomes—indicating that this age group is at risk for adverse maternal outcomes following birth. Similarly, a study conducted in southern Ethiopia revealed that mothers aged 35 years and above are six times more likely to experience an adverse maternal outcome than their counterparts (49). Older women face increased risks during pregnancy and childbirth mainly due to biological reasons that result in placental complications and high blood pressure (52). This underscores the need for enhanced monitoring and tailored care strategies for older pregnant women, including regular screenings for potential complications.

In this study, a poor wealth index classification, with an ARR = 3.81 (95% CI; 2.96, 4.89), showed an association with adverse maternal outcomes. A systematic review and meta-analysis report from sub-Saharan Africa revealed that mothers in the middle and rich wealth index groups tend to have a reduced risk of adverse maternal outcomes by 6% and 10%, respectively (53), compared to this study conducted in eastern Ethiopia. Similarly, a study conducted in northwestern Ethiopia also showed that women from poor households have a 3.3 times increased risk for adverse maternal outcomes (54). The wealth index serves as a proxy for socioeconomic status and is often linked to access to healthcare services, nutrition, and overall health literacy (55). Financial constraints and lack of transportation lead to delays in seeking or receiving appropriate medical attention during pregnancy and this increases the likelihood of complications and poor maternal outcomes (56). Thus, factors such as wealth index variation play a significant role in shaping these adverse maternal outcomes.

In this study, women using alcohol during pregnancy, with an ARR = 1.86 (95% CI; 1.32–2.62), had adverse maternal outcomes. Similarly, a study conducted in Ghana also revealed that mothers who drank alcohol during pregnancy experienced more than twofold more surgical site infections following CS birth than their counterparts (57). Similarly, a study conducted in Spain also showed that alcohol intake during pregnancy increased the risk of surgical site infections following CS birth compared to their counterparts (58). Pregnant women who consume alcohol during pregnancy may experience compromised immune function since alcohol can weaken the immune system, making it harder for the body to fight off infections postsurgery (59). Furthermore, alcohol may alter maternal metabolism (60) and lead to deficiencies in essential nutrients critical for wound healing, such as vitamin C, zinc, and protein (61).

Mothers who were diagnosed with admission danger signs, such as APH, pre-eclampsia, and obstructed labor (ARR = 2.75; 95% CI; 2.26–3.35), were found to be at greater risk of adverse maternal outcomes. A study conducted in Norway revealed that the risk of PPH was three times higher among pregnant women who had been diagnosed with severe pre-eclampsia at admission (62). These alarming statistics underscore the critical need for timely recognition and intervention. Similarly, a study conducted in southern Ethiopia showed that women who experienced complications of labor were 1.8 times more likely to experience postpartum hemorrhage (49). These findings collectively highlight that danger signs at admission can lead to significant delays in obstetric management, exacerbate the underlying condition, and ultimately result in adverse maternal outcomes (63). Thus, the systematic identification of these danger signs is crucial, as they often indicate life-threatening situations requiring immediate medical attention.

Having a labor duration of ≥24 h, with an ARR = 1.69 (95% CI; 1.00–2.85), was found to be a predictor of adverse maternal outcome. Similarly, studies conducted in Addis Ababa, Ethiopia, and Oslo also revealed that mothers who experienced prolonged labor had a more than twofold greater risk of experiencing PPH than their counterparts (64, 65). Prolonged labor can endanger women by increasing the risk of severe maternal complications, including postpartum hemorrhage, uterine rupture, and infection (66). Furthermore, extended labor duration can result in uterine atony (failure of the uterus to contract effectively), retained placenta, and other complications that contribute to excessive bleeding after delivery (67). This study conducted in eastern Ethiopia found that a vaginal delivery significantly contributes to reducing adverse maternal outcomes by 42% with an ARR = 0.58 (95% CI; 0.49–0.68). Similarly, a multicountry survey showed that, compared to vaginal birth, CS birth was associated with an increased risk of maternal intensive care unit admission and maternal near miss (68). Studies indicate that both cesarean deliveries (CDs) and operative vaginal deliveries are associated with increased risk of severe adverse maternal outcomes compared to vaginal deliveries. For instance, women undergoing primary cesarean sections have an adjusted odds ratio (aOR) of 10.59 for severe maternal outcomes, as compared to women who deliver vaginally (69). The vaginal mode of delivery is associated with lower risks of complications compared to CS. The natural process of vaginal birth promotes better uterine contractions postdelivery, which helps prevent conditions such as uterine atony and postpartum hemorrhage (70).

Folic acid is very useful for pregnant women to support the rapid growth of the placenta and fetus, and it helps to prevent anemia (71). In this study conducted in eastern Ethiopia, receiving folic acid during pregnancy reduced the occurrence of adverse maternal outcomes by 38%, with an ARR = 0.47 (95% CI; 0.38–0.57). Similarly, a systematic review and meta-analysis conducted in Pakistan on folate supplementation during pregnancy and its effect on maternal anemia and birth outcomes revealed that folate supplements taken during pregnancy reduce the risk of anemia in mothers by 69%, but it did not show a reduction in PPH (72). Furthermore, another systematic review and meta-analysis was conducted in high-, low-, and middle-income countries (Hong Kong, China, and Tanzania), which revealed that folate intake during pregnancy reduces the risk of anemia by 50% (73). Folic acid helps in the production of healthy red blood cells in the mother, which are essential for carrying oxygen throughout the body (74). The implications of these findings are significant for maternal health strategies. The substantial reduction in adverse outcomes associated with folic acid supplementation emphasizes the need for healthcare providers to promote its use among pregnant women.

Partners provide essential emotional backing, which can help reduce stress and anxiety levels in pregnant women and contribute to better maternal health outcomes (75). In this study conducted in eastern Ethiopia, partner support during pregnancy reduced the occurrence of adverse maternal outcomes by 30%, with an ARR = 0.70 (95% CI; 0.59–0.83). Partner support reduces stress during pregnancy (76), which can lead to a reduction of postpartum eclampsia (77).

## Strength and limitations

We employed appropriate statistical methods, such as multivariable Poisson regression with robust standard errors, to control for confounding variables, strengthen the validity of the findings, and accurately estimate risk ratios associated with adverse maternal outcomes. In addition, the study's multicenter design enhances its generalizability, as it encompasses a diverse population across various healthcare settings.

The study is not without some limitations. The short follow-up period and not following women after discharge from the hospital may overlook delayed or long-term maternal health complications. Therefore, we could not monitor outcomes throughout the puerperium and may have missed events after discharge, such as postpartum recovery, mental health challenges, and long-term maternal morbidity. Furthermore, variability in clinical practices across different healthcare institutions could affect how maternal care is delivered, leading to inconsistencies in outcomes. Such variability may result in differences in the management of complications during labor and delivery, which can exacerbate the risk of adverse maternal outcomes in the future as well. The self-reporting of substance use and recall bias were also significant limitations in this study.

## Conclusion

Almost one in six pregnant women experienced adverse maternal outcomes, and the overall rate of adverse maternal outcomes in eastern Ethiopia was determined to be moderate when compared to the WHO projections for lower- and middle-income countries (78). Anemia was the most prevalent complication, followed by postpartum hemorrhage. Several factors were found to be significantly associated with these adverse outcomes. Folic acid intake, vaginal mode of delivery and having partner support during pregnancy were identified as protective factors.

In contrast, a lower wealth index classification, maternal age older than 35 years, alcohol use during pregnancy, duration of labor longer than or equal to 24 h, and the presence of danger signs at admission were associated with an increased risk of adverse maternal outcomes. Addressing socioeconomic disparities, promoting folic acid supplementation, increasing partner support during pregnancy, closely monitoring high-risk pregnancies using targeted intervention programs, such as targeted education and empowerment programs, and strengthening community health worker programs will help to address socioeconomic disparities and improve the early detection and management of danger signs during pregnancy. These measures will aid in averting the occurrence of, and significantly reduce, adverse maternal outcomes.

## Data Availability

The raw data supporting the conclusions of this article will be made available by the authors, without undue reservation.
